# Synthesis of Various Size Gold Nanoparticles by Chemical Reduction Method with Different Solvent Polarity

**DOI:** 10.1186/s11671-020-03370-5

**Published:** 2020-07-02

**Authors:** Mohamed Hasaan Hussain, Noor Fitrah Abu Bakar, Ana Najwa Mustapa, Kim-Fatt Low, Nur Hidayati Othman, Fatmawati Adam

**Affiliations:** 1 Faculty of Chemical Engineering, Universiti Teknologi MARA, 40450 Shah Alam, Selangor Malaysia; 2 Faculty of Applied Science, Universiti Teknologi MARA, Tapah Campus, 35400 Tapah Road, Perak, Malaysia; 3 Faculty of Chemical and Process Engineering Technology, Universiti Malaysia Pahang, Lebuh Raya Tun Razak, 26300, Gambang, Pahang, Malaysia

**Keywords:** Gold nanoparticles, Chemical reduction, Solvent polarity index, Ethanol, PVP

## Abstract

Complicated and strict protocols are followed to tune the size of gold nanoparticles (GNPs) in chemical synthesis methods. In this study, we address the polarity of solvents as a tool for tailoring the size of GNPs in the chemical reduction method. The effects of varying polarity index of the reaction medium on synthesizing gold nanoparticles by chemical reduction method have been investigated. Ethanol as a polar solvent, ethanol–water mixture as reaction medium, L-ascorbic acid as reducing agent, and polyvinylpyrrolidone as stabilizer were used to synthesize GNPs. The polarity index of the reaction medium was adjusted by changing the volume ratio of ethanol to water. UV–Vis, dynamic light scattering (DLS), and transmission electron microscopy (TEM) characterizations reveal that the growth of nanoparticles was gradually increased (~ 22 to 219 nm hydrodynamic diameter) with decreasing value of polarity index of the reaction medium (~ 8.2 to 5.2). Furthermore, the high polarity index of the reaction medium produced smaller and spherical nanoparticles, whereas lower polarity index of reaction medium results in bigger size of GNPs with different shapes. These results imply that the mechanistic of the growth, assembly, and aggregation phenomena of ligand or stabilizer-capped GNPs strongly rely on the polarity of solvent molecules. Using the proposed methodology, wide size range of GNPs with different morphology sizes can be synthesized by simply modulating the volume percentage of organic solvent in the reaction medium.

## Introduction

Gold nanoparticles (GNPs) are recognized as a potential candidate in many areas of science and engineering applications including medical therapy [1], drug delivery [2], chemical sensing [3, 4], catalysation [5], and electronic [6] applications due to the size and shape-dependent surface plasmon resonance (SPR) [7], affinity with organic species and high electrical conductivity properties [8] of GNPs. Considering exponentially increasing demand of GNPs, far more attention is given to synthesize monodisperse nanoparticles with controllable size and morphology. Numbers of design principles have been proposed to control the properties of GNPs by incorporating different reactants, stabilizing agents or ligands [9], reaction conditions including temperature, pH, and concentration [4], and dispersed medium (such as different types of solvent) [10].

In chemical synthesis of GNPs, Turkevich method is a promising method compared to others. In Turkevich method, Au^3+^ ions are reduced by a mild reducing agent such as citrate [11], ascorbic acid [12], and tannic acid [13] in an aqueous medium. In this process, relatively small size and bio-compatible GNPs are produced. However, the main drawback of this method is highly controllable process protocol (temperature, concentration, and pH) that must be strictly followed to produce monodisperse particles with desirable sizes. Furthermore, in a pure aqueous medium, labeling of GNPs by organic drug molecules and surface modification with different ligands are difficult due to the less solubility and hydrophobicity of the organic component in water [14]. Thus, attention is given to overcome these limitations of Turkevich method by optimizing the reaction medium which significantly control the properties of solvent.

Solvent plays an important role in nanoparticle growth and assembly in colloidal synthesis process. Interaction between the nanoparticle surface and solvent molecules or interaction between the solvent molecules and ligand molecules considerably influences the final particle size and morphology [15, 16]. Generally, in absence of the passive ligands or capping agent, the strength of the electrical double layer that controls the particle growth is dominantly governed by the nature of solvent molecules. In high polarity index of dispersed medium, large amount of charged ions are adsorbed by nanoparticles surface, whereby a strong electrical double layer forms around the colloidal particles [17]. As a result, the zeta potential of particle increases, and the particles are prevented from aggregation by repelling each other. However, the surface charge around the nanoparticles can be manipulated by the solvent polarity, and the interaction between the particles is controlled prior to optimize the size and shape [18]. For instance, Song et al*.* produced 1–6 nm range of thiol-capped GNPs in different polarity of organic solvent [19]. Although few works have been proposed for optimizing the size of GNPs in different polarity of solvent, the size of final particles lies between a small range (less than 20 nm) which cannot completely satisfy the major applications of GNPs such as for therapeutic and drug delivery.

On the other hand, in presence of ligands in the reaction medium, interactions between solvent molecules and ligands considerably affect the growth and assembly of nanoparticles. Similar studies show that the nonaqueous medium solvate organic ligand molecules in the colloidal solution protect nanoparticles from further growth and aggregation to a great extent [20, 21]. However, under certain circumstances such as at higher concentration, higher molecular weight and high length of ligands may cause aggregation or high growth in colloidal nanoparticles. Notably, different swelling and elongating rate of ligand molecules in different polarity of the solvents easily trigger the nanoparticle growth and assembly in colloidal synthesis [22]. However, this negative effect can be turned as a tool to grow nanoparticles with controllable size. More than the direct involvement of solvent in nanoparticle growth, the concept of governing the properties of ligands using solvent may allow to synthesize GNPs with wide range of size scale.

Considering aforementioned proposition, this study proposes a method to optimize GNPs size and morphology by controlling the ligand or stabilizer and the surface charges using polarity index of the reaction medium as tool. In this study, GNPs was synthesized via chemical reduction method using L-ascorbic acid as reducing agent and PVP as stabilizing agent. In comparison to water molecules, polarity index of ethanol (selected polar organic solvent in this study) is less. Therefore, the polarity index of the reaction medium was manipulated by adding ethanol to water in various volume ratios. Unlike the conventional Turkevich method, it is expected to synthesize GNPs with wide range of preferable size GNPs using this proposed technique in a single step with the minimum protocol.

## Methods

### Material

HAuCl_4_∙3H_2_O (≥ 99% trace metal basis), crystalline L-ascorbic acid, polyvinylpyrrolidone (PVP) K60, and sodium hydroxide (NaOH) pellets were purchased from Sigma-Aldrich (Missouri, USA). Ethanol (99.90% assay) was obtained from J-Kollin Chemicals (UK), and double distilled water (ddH_2_O) was obtained from the laboratory (Faculty of Chemical Engineering, UiTM, Malaysia).

### Preparation of Gold Nanoparticles (GNPs)

Colloidal GNPs were prepared using an ascorbic acid incorporated modified Turkevich approach [23]. In this method, L-ascorbic acid was used as a reducing agent while polyvinyl pyrrolidine (PVP) as a stabilizer. The reducing agent solution was prepared by dissolving L-ascorbic acid in absolute ethanol or 20%, 50%, and 80% volume ratios of ethanol to water binary solvent mixture. The total volume of water that was added to the ethanol to water binary solvent mixture was determined according to Eq. (1). In addition, PVP was directly dissolved into the L-ascorbic acid/ethanol to water solvent mixture to the final concentration of 1% (w/v) under low decibel sonication. Finally, pH of reducing agent solution was adjusted to 10.5 from its initial value by adding 2 M NaOH in drop-wise.

1 $$ Volume\ percentage\ of\ water\ in\ solvent\ mixture\ \left(\%\right)=\frac{V_w+{V}_{GC}}{V_R}\times 100\kern0.5em $$

where volume of water is *V*_*W*_, volume of gold chloride is *V*_*GC*_, and total volume of the reaction mixture *is V*_*R*_*.*

GNP forming reaction mixture was prepared by rapidly injecting 5 mM HAuCl_4_ aqueous solution to the reducing agent solution. The total volume of the reaction mixture was maintained at 20 ml of HAuCl_4_ and L-ascorbic acid solutions. The initial concentration of the HAuCl_4_ and L-ascorbic acid in the final reaction mixture was set as 0.15 mM and 1.5 mM respectively with molar ratio of HAuCl_4_ to L-ascorbic acid of 1:10. The reaction mixture was vigorously stirred with 800 rpm for 30 min at ambient temperature, and the resultant GNP colloid was filtered using Whatman laboratory filter paper before storage at 4 °C to avoid a continuous reaction.

### Characterization and Instrumentation

#### UV–vis Characterization

UV visible extinction spectra of each GNPs sample were measured at ambient temperature (25 °C) using an Agilent Cary 60 UV–Vis Spectrophotometer. Maximum surface plasmon resonance (SPR) wavelength of the resultant GNPs was determined from the obtained UV–Vis data to compare the particle size.

#### Particle size distribution and zeta potential

Mean particle size, size distribution, poly-dispersity index (PDI), and zeta potential of GNPs were measured using a Malvern Zetasizer nanoZS instrument. The PDI value was calculated from intensity particle size distribution (PSD) graphs of dynamic light scattering (DLS) measurements using Eq. (2) [24]:

2 $$ PDI={\left( standard\ deviation/ mean\ particle\ size\right)}^2 $$

#### Polarity Index Analysis of Mixed Solvent

The polarity of the reaction medium was optimized by mixing different volumetric ratios of water and organic solvent. Further, the net polarity index of organic solvent-water resultant mixture, *P*′, can be given by Eq. (3) [25].

3 $$ {P}^{\prime }=\sum {p}_i^{\prime }{\varnothing}_i $$

where, ${p}_i^{\prime }$is the polarity index of solvent *i* , and ∅_*i*_ is the volume fraction of solvent *i* in the mixture. The polarity index of distilled water and absolute ethanol were 9.0 and 5.2, respectively.

#### Transmission Electron Microscopy (TEM) and Particle Size Distribution

GNP samples were sonicated prior to TEM measurement. Droplets of the sonicated suspensions/colloids were then dropped onto 200 mesh Formvar copper grid. The grid was placed in a “single tilt” sample holder following by inserting into a 200-kV FEI, Tecnai G2 20 Twin Transmission Electron Microscope for imaging. In addition, quantitative analysis of particle size and size distribution from TEM images were analyzed using the ImageJ image processing software.

## Results and Discussion

**Fig. 1:**
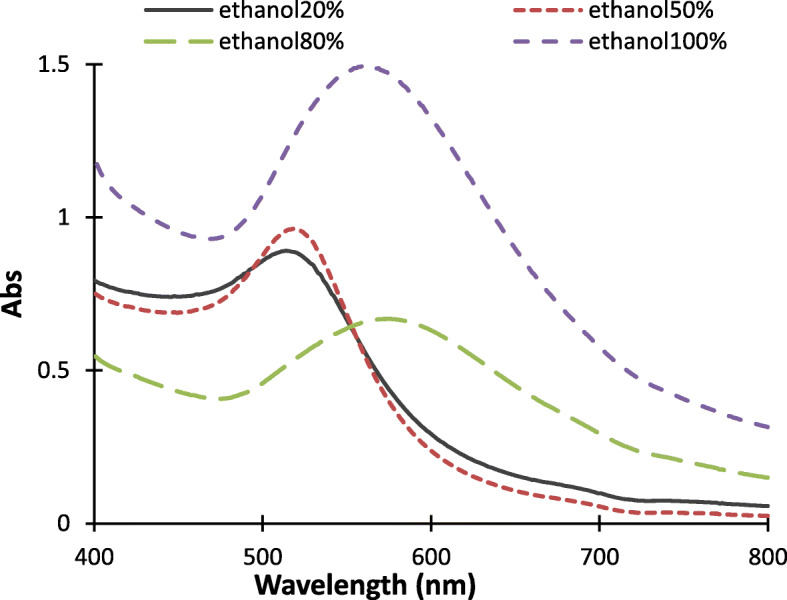
UV–Vis spectra of GNPs in 20%,50%,80% and 100%volume percentage of ethanol to water binary solvent mixture

Figure 1 depicts the UV–Vis spectra of synthesized colloidal GNPs in different volumetric ratios of ethanol to water solvent mixture. Typically, maximum SPR absorption wavelength (λ_m_) of GNPs is size and shape dependent [26, 27]. As shown in Fig. 1, maximum λ_m_ of the synthesized colloidal GNP shift to the right which implicits the size of synthesized GNPs increased with an increasing volumetric ratio of ethanol in water. The maximum λ_m_ of the colloidal GNPs synthesized in reaction mixtures containing ethanol with 20% and 50% volumetric percentage appears in the shorter region of absorption wavelengths (514 nm and 520 nm) which implicit that small sizes of GNPs were produced in a low volume percentage of ethanol. In addition, the maximum λ_m_ of GNPs synthesized in ethanol to water with 80% volumetric percentage or absolute ethanol shifted to the higher wavelength regions at 575 nm and 561 nm respectively. These shifts indicate the formation of larger size and broad trend of the graphs that imply the uneven shapes of GNPs.

**Fig. 2:**
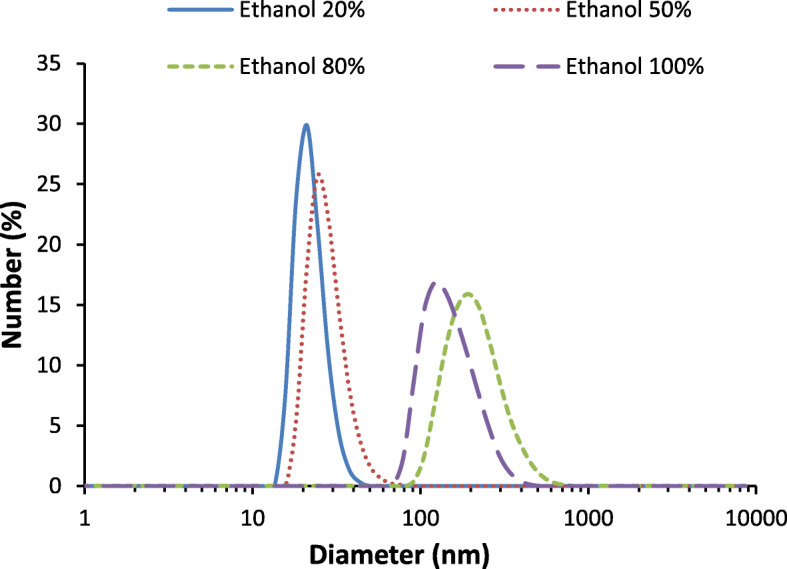
DLS size distribution trends of GNPs particles in 20%, 50%, 80%, and 100% volume percentage of ethanol to water binary solvent mixture

The synthesized GNPs in different volumetric percentage of ethanol to water mixtures with different polarity indexes were quantified using DLS, and the obtained size distribution of GNPs are shown in Fig. 2. In addition, the mean particle size, PDI values of GNPs, and polarity indexes of ethanol–water mixtures of produced GNPs are summarized in Table 1. Figure 2 shows that the DLS size distribution increases with increasing volumetric percentage of ethanol. The mean hydrodynamic diameters of GNPs in solvent mixtures containing absolute ethanol, 80%, 50%, and 20% ethanol to water volumetric percentage, were 154 ± 56.7, 219 ± 84.9, 28 ± 10.5, and 22 ± 4.6 nm respectively (c.f. Table 1). These DLS results are similar to the UV–Vis findings of this study which small particles were produced in high polarity index of ethanol–water mixture and vice versa.

**Table 1:** Hydrodynamic mean diameter and PDI value GNPs in different volumetric percentage of ethanol obtained by DLS method and calculated polarity index of various ratio of organic solvent-water medium

Solvent-aqueous volume percentage	Hydrodynamic mean diameter of GNPs (nm)	Poly dispersity index (PDI)	Polarity index of solvent mixture
20% ethanol–water solution	22 ± 4.6	0.40	8.24
50% ethanol–water solution	28 ± 10.5	0.12	7.10
80% ethanol–water solution	219 ± 84.9	0.17	5.96
100% ethanol	154 ± 56.8	0.15	5.20

**Fig. 3:**
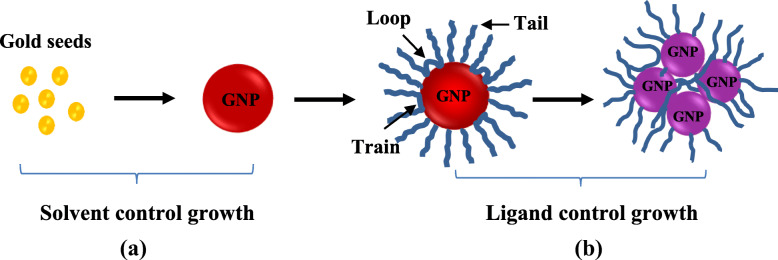
Growth stages of GNPs in colloidal solution in presence of long polymer chain ligand molecules where the growth of GNPs is governed by (**a**) solvent molecules and (**b**) ligand molecules

The calculated polarity index values of different volume percentage of ethanol to water solvent mixtures are given in Table 1. The polarity index of binary solvent mixture decreases with an increasing volumetric percentage of ethanol (organic solvent) in the solvent mixture. Herein, UV–Vis and DLS results of this study reveal that the smaller size of GNPs was produced in high polarity index of reaction medium, whereas bigger size of GNPs was produced in low polarity index of reaction medium. It has been well studied that both solvent and ligands play an important role in controlling the nanoparticle growth and assembly in colloidal nanoparticle synthesis process. During nanoparticle formation, solvent molecules and ligand molecules control and slow down the particle growths by blocking the surface binding sites. However, in certain conditions such as different polarity of solvent mixture, the solvent molecules and ligand molecules may also trigger the growth and assembly of nanoparticles in colloidal solutions. In agreement with this statement, UV–Vis and DLS results show that the growth and assembly of PVP-capped GNPs increased with increasing volume percentage of ethanol in binary solvent mixture. To validate these findings, corresponding mechanism for the growth of PVP capped GNPs in ethanol–water binary solvent mixture can be interpreted in two stages (as shown in Fig. 3) which are solvent caused growth and ligand-induced growth.

In the first stage, after nucleation of monomers, the growth of GNPs was governed by the solvent molecules. In colloidal solution, high polar solvent molecules distribute high surface charge on the nanoparticle surface and built a strong electrical double layer that prevents particles from further growth, whereas low polarity index solvent molecules formed a weak electrical double layer around the particles resulting in higher growth or aggregation [28]. The correlation between the surface charge and polarity index of reaction can be explained using Poisson’s equation given in Eq. (4) [29].

4 $$ \varepsilon {\varepsilon}_o\frac{d^2\psi (x)}{d{x}^2}=-\rho (x)\kern4em $$

where *ρ* is the charge density, *ψ* is the electrical potential, *ε* is the permittivity of vacuum, *ε*_*o*_ is the dielectric constant, and *x* is the distance between the particle surface.

According to Eq. (4), charge density on the nanoparticle surface (*ρ*) is proportional to the dielectric constant (*ε*_*o*_) of the surrounding which is governed by the polarity index of medium. In this study, the polarity index value of corresponding solvents can be ordered as water > ethanol. Therefore, dispersed medium which has higher volumetric percentage of water can offer higher surface charge to the GNP surface due to high polarity. Besides, ascorbic acid acts as a reducing agent to donate the electron to the metal salt to form a gold nanoparticle. Ascorbic acid has a higher solubility in pure water than absolute ethanol, thereby more hydrogen bonding establishment form with water molecules. Also, the higher solubility could promote more free electron transfer for fast nucleation of Au^0^ monomers that lead to slow growth of GNPs [30]. Due to these facts, the growth rate of GNPs was inversed to the polarity index value of ethanol–water solvent mixture.

**Fig. 4:**
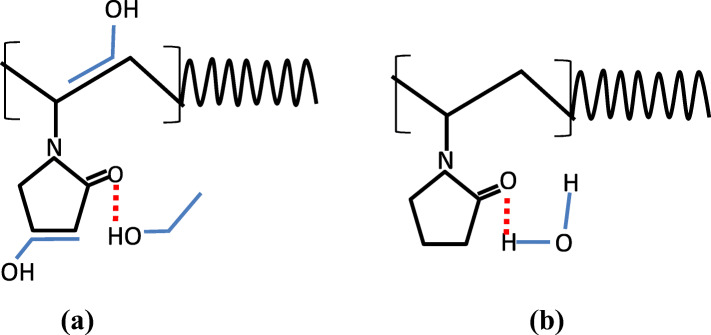
(**a**) Interaction between ethanol molecules and PVP molecules and (**b**) Interaction between H_2_O molecules and PVP molecules

In the second stage, it was hypothesized that the growth and assembly of GNPs were predominantly governed by ligand molecules (PVP) (c.f. Fig. 3(b)). In the presence of ligand molecules in the colloidal solution, the ligands are adsorbed or covalently attached with nanoparticle surface and prevent the nanoparticles from further growth and assembly by controlling the interfacial energy between the particle surface and solvent or creating repulsive forces between similar ligand-coated particles [28]. However, if adsorbed ligand molecules have high concentration, long polymer chain, or high molecular weight, they form tail, train, and loop into the colloidal solution. As depicted in Fig. 3 (b), these protruding tails, trains, and loops of the ligand chains bridge the nanoparticles and induce flocculation in colloids [31]. This bridging flocculation in nanoparticles may promote the secondary seed-mediated growth in nanoparticles, whereby particles can be easily grown. In this study, 1% (w/v) of PVP was used to stabilize the GNPs in colloidal solution. PVP is an amphiphilic molecule which has a hydrophilic head (which consists of C = O and N species) and highly hydrophobic hydrocarbon back chain. In polar organic solvent, organic molecules have a great affinity with both carboxylic group and backbone hydrocarbon tail of PVP as depicted in Fig. 4 (a). However, merely in an aqueous medium, water molecules can only bind with the carboxylic head of PVP via hydrogen bond, and hydrophobic tails remain in the suspension independently as depicted in Fig. 4 (b). Thus, PVP molecules are solvated and swelled in an organic solvent to a higher extent than pure aqueous medium [21]. As evidence, Guettari et al. investigated the behavior of PVP polymer in different volumetric percentages of ethanol to water solvent mixture. Experimental results with effective solvent interaction with polymer (ESIP) modeling of this work confirm that the hydrodynamic radius and polymer-polymer interaction of PVP molecules increase with increasing molar fraction of ethanol [32]. This increasing globular size of the PVP molecules in higher volume percentage of ethanol enhance the bridging of GNPs results high growth or assembly. Therefore, it was concluded that highly extended 1% (w/v) of PVP chains flocculated the particles and formed the different shape of assembly or aggregation of GNPs which lead high growth in a high volumetric ratio of ethanol [33–35].

**Fig. 5:**
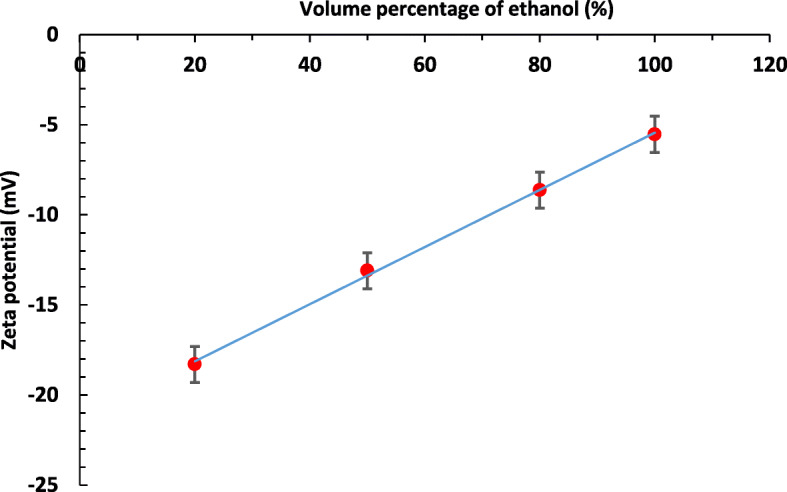
Zeta potential values of PVP stabilized GNPs in 20%, 50%, 80% and 100% volume percentage of ethanol to water binary solvent mixture

Furthermore, the influence of ligand molecules on nanoparticle growth and assembly in varying polarity index of ethanol–water solvent mixture was analyzed by the zeta potential of the resultant GNPs. The zeta potential values of GNPs that were synthesized in a different volumetric ratio of ethanol to water are shown in Fig. 5. The obtained results show that the zeta potential values of PVP stabilized GNPs decrease with increasing volume percentage of ethanol. In general, the polymer displaces the slip plane of the electrical double layer of the particles which can change the zeta potential value. The changes in the value of zeta potential depend on the interfacial surface charge and the amount of the adsorbed polymer [22]. Similar studies found that the zeta potential value of fully PVP capped GNPs is about - 6 mV [23, 36]. Besides, this negative zeta potential value may increase with decreasing adsorption amount PVP on GNP surface [22]. Therefore, the obtained lowest value of zeta potential (-5.53 mV) in absolute ethanol indicates that the synthesized GNPs were fully surrounded by PVP molecules. On the other hand, zeta potential values decreased with increasing volumetric ratio of ethanol implicit that the PVP adsorbed to a high degree in a high volumetric ratio of organic solvent (ethanol).

**Fig. 6:**
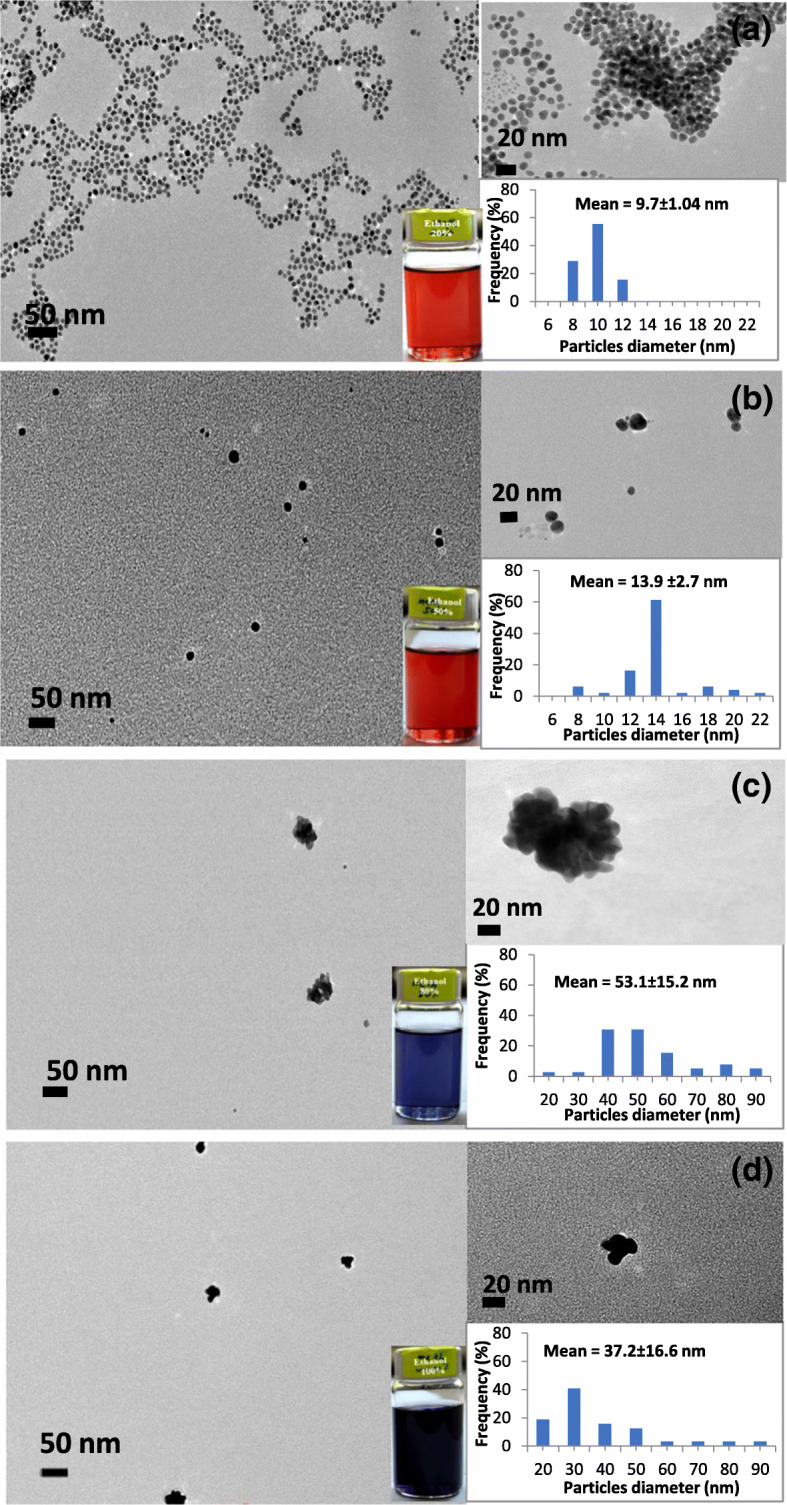
Colors, TEM images, and size distribution of the produced GNP suspension in various volume percentage of ethanol to water with (**a**) 20%, (**b**) 50%, (**c**) 80%, and (**d**) 100% ethanol

TEM images of the synthesized GNPs in different polarity index of ethanol to water solvent mixtures are shown in Fig. 6. Figures 6 (a) and (b) show that 9.7 nm and 13.9 nm of nearly spherical nanoparticles were produced in 20% and 50% volumetric percentage of ethanol to water solvent mixture respectively. On the other hand, Fig. 6 (c) shows the typical images of GNPs in 80% volumetric percentage of ethanol to water mixture. TEM images reveal that irregular shape [37] and bigger size (about 53.1 nm) of GNPs were formed in 80% volumetric percentage of ethanol solution, and these particles were aggregated in the colloidal solution. Similarly, 37.2 nm mean diameter of relatively larger and irregular shape of GNPs was produced in absolute ethanol as well. These results comply to the previous UV–Vis and DLS results of this study, in which the bigger size of particles was formed due to low value of the polarity index of reaction medium that consequent the growth of particles and assembly caused by the highly extended PVP polymer chain in low polarity index of ethanol–solvent mixture.

It is worthwhile to inform that the size of GNPs in 80% volumetric percentage of ethanol to water is larger than the size of GNPs in absolute ethanol.

**Fig. 7:**
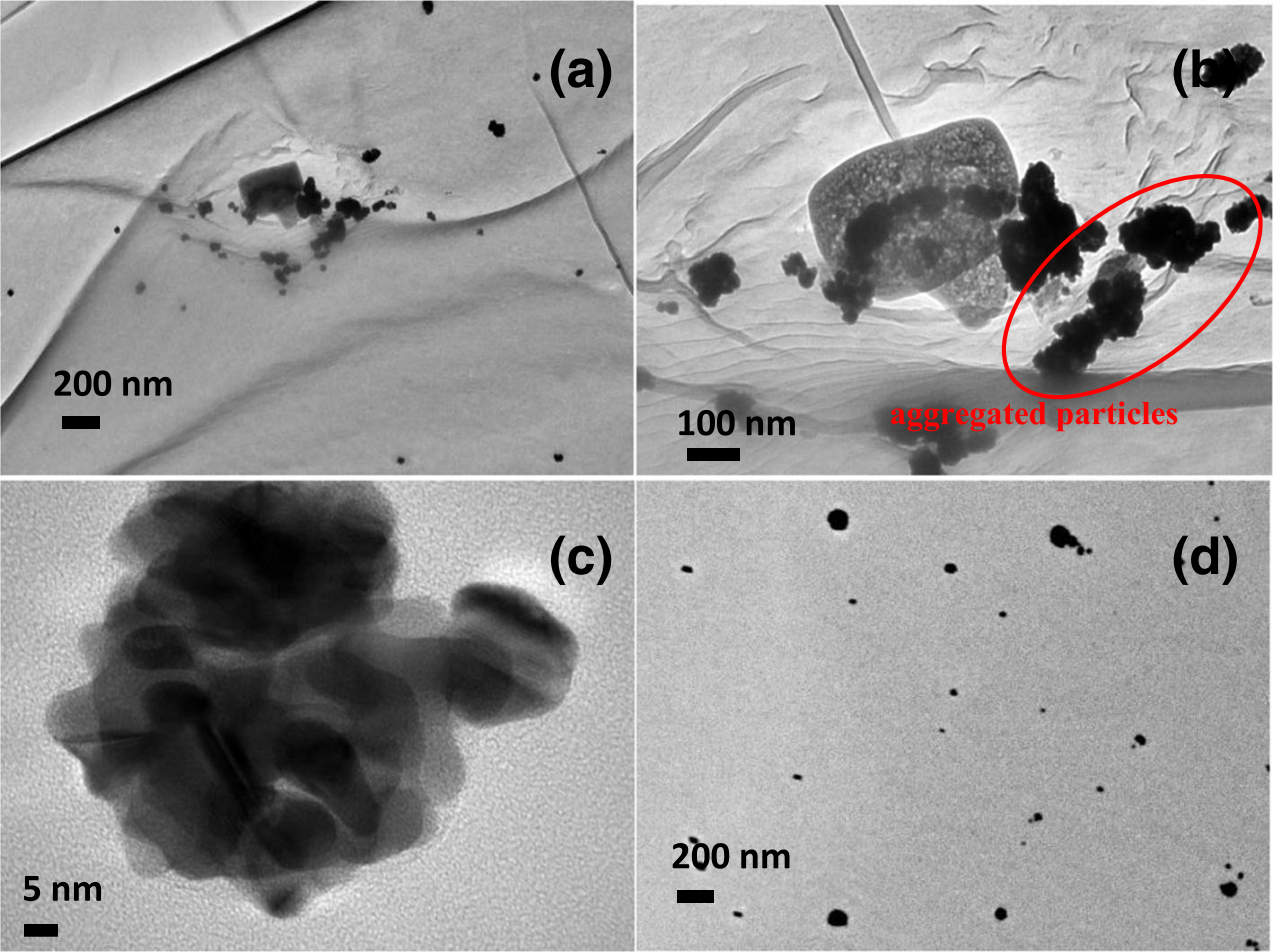
TEM images of GNPs (**a**), (**b**) and (**c**) aggregated particles in 80% volumetric percentage of ethanol (**d**) discrete particles in 100% ethanol

Figures 7 (a) and (d) compare the TEM images of GNPs in 80% volumetric ratio of ethanol to water and absolute ethanol respectively. The GNPs were aggregated as clusters (Fig. 7(a–c)) and aligned linearly (Fig. 7 (a) and (b)) in 80% volumetric ratio of ethanol, whereas GNPs in absolute ethanol remained as discrete particles with less aggregation (Fig. 7d). We hypothesized that the composition of 80% ethanol to water rapidly increased the surface energy of GNP primary particles due to the asymmetric interaction of water and ethanol molecules with PVP polymer chain and nanoparticle surfaces. Therefore, particles aggregated by oriented-attachment mechanism formed larger size of nanoclusters in order to minimize this surface energy [38, 39]. Furthermore, the linear alignment of GNPs in 80% ethanol to water attributed to the dipole-dipole interaction of the particles which was resulted by the asymmetric interaction of water and ethanol molecules with PVP polymer chain [40]. In addition, it was observed that the mean hydrodynamic diameter of GNPs obtained using DLS was higher than the size calculated from TEM images. In this study, GNPs were synthesized in excessive amount of PVP polymer solution. Therefore, the DLS technique measured not only the diameter of the particles but also the capping polymer layer with elongated polymer tail, train, and loop. Moreover, DLS may measure the size of flocs instead of individual particles (e.g., 80% ethanol to water GNPs sample). Consequently, the average size of GNPs measured using DLS was higher than using TEM.

## Conclusion

In this study, the synthesize of size selective GNPs using polarity of organic solvent as a variable has been discussed. Influence of solvent polarity in GNP growth has been investigated by synthesizing PVP capped GNPs in ethanol and ethanol–water mixtures in L-ascorbic acid. UV–vis spectra and DLS measurements confirmed that particle size increases with decreasing polarity index of solvent. Based on these results, the growth of GNPs was controlled in two stages during the chemical reduction process. The particle growth was initially controlled by the solvent molecules by forming a strong double layer around the nanoparticle. Then, the assembly and stability of GNPs are governed by the stabilizer or ligand molecules in the second stage. However, the growth of GNPs increases with decreasing polarity index of the reaction medium in both stages. The final colors of suspended GNPs and TEM images implicit the morphologies of the produced GNPs. Notably, high polarity of the solvent mixture resulted in spherical shape GNPs, and low polarity index environment resulted in irregular shape of GNPs. This investigation addressed a new approach to synthesize various size and shape of GNPs in a single step by taking advantage of solvent polarity index-dependent particle growth and assembly.

## Data Availability

All data generated or analyzed during this study are included in this study. The raw dataset obtained and analyzed during the experimental work is available from the corresponding author on reasonable request.

## Abbreviations

GNPs: Gold nanoparticles
NPs: Nanoparticles
PVP: Polyvinylpyrrolidone
TEM: Transmission electron microscopy
DLS: Dynamic light scattering
PDI: Polydispersity index
